# Surgery in Oligometastatic Pancreatic Cancer: Narrative Systematic Review

**DOI:** 10.3390/cancers18111699

**Published:** 2026-05-23

**Authors:** Ulrich Ronellenfitsch, Rosa Klotz, Jörg Kleeff, Christoph Michalski, Artur Rebelo

**Affiliations:** 1 Department of Visceral, Vascular and Endocrine Surgery, Medical Faculty of the Martin-Luther-University Halle-Wittenberg and University Hospital Halle (Saale), 06120 Halle (Saale), Germany; joerg.kleeff@uk-halle.de (J.K.); artur.rebelo@uk-halle.de (A.R.); 2 Department of General, Visceral and Transplantation Surgery, Heidelberg University Hospital, 69120 Heidelberg, Germany; rosa.klotz@med.uni-heidelberg.de (R.K.); christoph.michalski@med.uni-heidelberg.de (C.M.)

**Keywords:** pancreatic ductal adenocarcinoma, oligometastatis, metastasectomy, surgical resection, multimodal therapy

## Abstract

Background: Pancreatic ductal adenocarcinoma (PDAC) carries a poor prognosis, particularly in metastatic disease where surgical resection is traditionally not recommended. However, the concept of oligometastatic disease—characterized by limited metastatic spread with a presumed more favorable oncological prognosis—has prompted reconsideration of metastasis-directed therapies, including surgery, in carefully selected patients. Methods: A selective literature review was conducted using PubMed to identify studies reporting surgical treatment for oligometastatic PDAC, including synchronous or metachronous metastases. Studies were included if they reported outcomes after resection of the primary tumor and/or metastatic lesions in patients with limited metastatic burden. Results were presented in a narrative way. Results: Retrospective studies suggest that surgical resection of the primary tumor and metastases may be feasible, safe and associated with prolonged survival in selected patients with oligometastatic PDAC. Favorable prognostic factors across studies include response to systemic chemotherapy prior to resection, low tumor marker levels, limited number of metastases, and the possibility of achieving complete macroscopic resection. Patients with isolated pulmonary metastases appear to have particularly favorable outcomes. However, existing evidence is derived exclusively from retrospective analyses and is subject to considerable selection bias. Conclusions: Current evidence indicates that surgery for oligometastatic PDAC may benefit highly selected patients within a multimodal treatment strategy. The results of ongoing prospective and randomized clinical trials are expected to clarify the role of surgery in this setting. Until these results become available, treatment decisions should be individualized and made within multidisciplinary tumor boards.

## Simple Summary

Pancreatic cancer that has already spread is usually treated without surgery because outcomes are poor. However, some patients have only a small number of metastases, a situation called oligometastatic disease. In these selected cases, surgery to remove the main tumor and metastatic lesions may be possible as part of a combined treatment strategy. This review summarizes the current evidence on surgery for oligometastatic pancreatic cancer. Retrospective studies suggest that surgery can be safe and may be linked to longer survival in carefully selected patients, especially when metastatic spread is limited, chemotherapy works well before surgery, tumor marker levels are low, and complete removal of visible disease is possible. Patients with isolated lung metastases may have particularly favorable outcomes. At present, however, the available data are based only on retrospective analyses and are prone to selection bias. Ongoing prospective and randomized trials are expected to clarify which patients may truly benefit from surgery.

## 1. Introduction

Pancreatic cancer, i.e., pancreatic ductal adenocarcinoma (PDAC), is the twelfth most common cancer worldwide and ranks ninth in cancer mortality. For 2021, age-standardized pancreatic cancer incidence worldwide was estimated 5.9/100,000, corresponding to 508,533 new cases. Age-standardized mortality was 5.9/100,000, corresponding to 505,752 annual deaths. It is estimated that until 2044, the number of both incident cases and deaths will be above 875,000 [1]. Five-year survival of patients with PDAC across all stages is below 10%, and below 5% for patients in metastatic stages [2,3]. Traditionally, and still in the majority of treatment guidelines, the presence of metastatic disease has excluded patients from surgical resection, and palliative, non-operative treatment is recommended [4].

In oncology, there is the intriguing concept of “oligometastases”. The term was first coined by Hellman and Weichselbaum in 1995 in contrast to the theorems that malignant tumors either contiguously develop from a localized to a metastatic state, and once this state is reached tumors are aggressive and spread widely, or that most malignant diseases are primarily metastatic even if only the primary tumor is clinically apparent [5]. In both scenarios, local treatment of metastatic disease would be rendered moot with regard to prognosis and survival. Oligometastatic disease, in contrast, describes a state where tumors, as a result of a moderate “aggressiveness”, have a confined metastatic potential resulting in a spread to one or few distant locations, making patients amenable to local treatment with prognosis-improving or even curative potential [6]. There is no uniform definition of what exactly constitutes oligometastatic disease. Attempts for consensual definition have been made and take the overall number of metastatic lesions, the number of metastatic organs and the number of lesions per organ into account [7]. Commonly, situations with no more than five metastases in one or two organs are considered oligometastatic when it comes to possible local treatment. In PDAC, most definitions consider oligometastatic disease as when there are up to three metastases in one single site, usually either the liver or the lung, with exclusive hepatic metastases being much more common than exclusive pulmonary ones [8].

With the advent of more effective systemic therapies for PDAC offering both a chance of partial response to facilitate metastasectomy and of a “test-of-time” for the biological behavior of PDAC prior to it, comparable to concepts in colorectal cancer [9], metastasis-directed surgery in patients with oligometastatic PDAC has been increasingly applied over the last two decades. An important contributor has been the transition from gemcitabine monotherapy to modern combination regimens such as FOLFIRINOX and gemcitabine/nab-paclitaxel, yielding high objective response rates [10].

Early case reports and small series demonstrated the technical feasibility of resecting isolated synchronous or metachronous liver metastases in highly selected patients. Subsequent larger cohort studies, registry and multicenter analyses suggested that, in selected patients with liver-limited oligometastatic disease, surgical resection of both the primary tumor and metastases may be associated with prolonged survival compared with non-operative management or exploration alone. This signal was consistently observed in patients with favorable tumor biology, limited metastatic burden, good performance status, and treatment in high-volume centers. More contemporary retrospective studies refined the concept of patient selection by emphasizing response to neoadjuvant chemotherapy, biological surrogates of tumor aggressiveness, and the feasibility of achieving complete macroscopic resection, further supporting the hypothesis that a subset of patients with oligometastatic PDAC may derive meaningful survival benefit from surgery after effective systemic treatment. In this article, the use, feasibility and outcomes of surgery for oligometastatic PDAC will be reviewed.

## 2. Methods

A selective literature review was conducted in PubMed (from inception to 1 February 2026). The search term (((pancreatic cancer[MeSH Terms]) AND (metastases[MeSH Terms]) AND (surgery[MeSH Terms]))) was used. In addition, reference lists of selected publications were searched. Studies were included if the publication was in English and reported on outcomes of surgery for distant metastases of PDAC in at least ten patients if the disease was considered “oligometastatic” in the respective study, or fulfilled the criteria commonly used to define oligometastatic disease (no more than five metastases in one or two organs). Studies on both synchronous and metachronous treatment of the primary tumor and metastatic lesions were eligible. The Newcastle–Ottawa Scale for cohort studies was used for quality and risk-of-bias assessment of the single studies.

## 3. Summary of Evidence

The most important studies reporting on surgery for oligometastatic PDAC are summarized in Table 1. Quality and risk-of-bias assessment using the Newcastle–Ottawa Scale is presented in Table S1. One of the earliest studies was conducted by Shrikhande et al., who retrospectively analyzed 29 patients undergoing pancreatic resections along with metastasectomy [11]. Including patients with liver, peritoneal, and interaortocaval lymph node metastases, the study reported a 0% in-hospital mortality and 24.1% morbidity. Median overall survival reached 13.8 months, with notably improved outcomes (median overall survival of 27 months) in patients with interaortocaval lymph node metastases.

In a retrospective multicenter study including six high-volume European pancreatic centers, Tachezy et al. analyzed 69 patients with PDAC and synchronous liver oligometastases who underwent simultaneous resection of the primary tumor and liver metastases and compared them with a control group undergoing exploration without resection [12]. Median overall survival was significantly longer in the resection group (14 vs. 8 months). Subgroup analyses revealed that this survival advantage was driven primarily by patients with pancreatic head tumors (median overall survival 13.6 vs. 7 months), whereas no benefit was observed for tumors of the pancreatic body or tail. On multivariable analysis, surgical resection was the only independent predictor of overall survival (HR 2.04).

In a large single-center analysis from a high-volume pancreatic surgery unit, Hackert et al. evaluated prospectively collected data from 128 patients with PDAC who underwent curative-intent resection of the primary tumor and oligometastatic disease over a 12-year period [14]. Metastatic sites included the liver (*n* = 85) and extra-regional lymph nodes (*n* = 43), with patients stratified according to synchronous versus metachronous metastasectomy. Resections were performed with acceptable perioperative risk, with an overall morbidity of 45% and a 30-day mortality of 2.9%. Median overall survival was 12.3 months, comparable between liver and interaortocaval lymph node metastases. Long-term follow-up revealed 5-year survival rates of 8.1% after liver metastasectomy and 10.1% after interaortocaval lymph node resection.

Frigerio et al. provided a retrospective analysis on surgical resection following downstaging chemotherapy in metastatic PDAC [13]. Among 535 patients, 24 (4.5%) achieved significant tumor regression including complete radiologic disappearance of liver metastases after neoadjuvant therapy (mostly FOLFIRINOX). R0 resection of the primary was achieved in 88% of cases. The former metastatic bed in the liver was not resected. Median overall survival reached 56 months, and disease-free survival 27 months. Surgical morbidity was acceptable, with no perioperative deaths.

Kandel et al. conducted a case–control study evaluating outcomes of patients with oligometastatic PDAC treated with a combined modality approach including neoadjuvant therapy, metastasectomy and/or ablation, and resection of the primary tumor [16]. Patients undergoing surgery for metastatic disease (M1 surgery) were matched 1:3 by T and N stage to two control cohorts: patients with non-metastatic PDAC undergoing resection (M0 surgery) and patients with metastatic PDAC treated with chemotherapy alone (M1 no surgery). Median overall survival was 2.7 years in the M1 surgery group, compared with 2.0 years in the M0 surgery group and 1.0 year in the M1 no surgery group. After adjustment for ECOG performance status, M1 surgery patients demonstrated significantly improved survival compared with M1 no surgery patients and comparable survival M0 surgery patients.

Liu et al. conducted an analysis in the SEER database and identified patients with synchronous single-organ metastasis from PDAC for the years 2010–2014. Of these, 128 out of 6252 patients with liver metastasis underwent resection. Survival from diagnosis was 10.0 months versus 4.0 months for resected versus not resected patients (*p* < 0.0001) [17].

Bachellier et al. analyzed 92 patients undergoing synchronous resection of PDAC and liver metastases [18]. The study reported a median overall survival of 18.3 months from diagnosis and 12.7 months from surgery, with a 1-year survival of 70% but a stark decline at 3 years (10%) and no survivors at 5 years. Multivariable Cox analysis revealed that preoperative CA19-9 < 500 kU/L, R0 resection, and adjuvant chemotherapy were independent predictors of longer survival. Patients who received neoadjuvant treatment had a longer median overall survival from diagnosis (22.7 vs. 13.8 months).

Hank et al. reported on a cohort of 173 patients with metastatic PDAC operated on between 2006 and 2019. A total of 93 proceeded to resection while 80 underwent exploration only [15]. Median overall survival was significantly prolonged in patients who achieved complete pathological response in metastases (25.5 months) compared to those with residual disease (10.7 months) and non-resected patients (8.1 months). Adjuvant chemotherapy provided additional survival benefit, with median overall survival reaching 29.1 months in ypM0 patients who received adjuvant therapy. Multivariable analysis confirmed that metastasectomy after response to systemic therapy, CA19-9 levels, and time to resection were independent prognostic factors.

Nagai et al. published a retrospective cohort from a high-volume center [19]. A total of 47 patients underwent liver resection for metastatic PDAC between 2000 and 2019. Patients who received systemic chemotherapy followed by planned resection demonstrated a median overall survival of 38.1 months from diagnosis and 24.1 months post-surgery. In contrast, patients with incidental intraoperative liver metastasis who underwent unplanned resection had a shorter median overall survival of 8.7 months. Multivariate analysis identified preoperative chemotherapy and moderate to well-differentiated tumor grading as significant predictors of prolonged survival.

The recently completed retrospective ALTOPANC study included 155 patients with no more than five PDAC metastases to two organs who underwent resection of the primary tumor and simultaneous or staged metastasis-directed treatment, i.e., surgery, radiotherapy, or ablation. Metastatic sites included the liver (*n* = 71) and lung (*n* = 61). While median EFS and median OS in the entire population were 0.8 and 3.4 years, respectively, a score based on CA 19-9, number and location of metastasis was prognostic [20].

With the exception of ALTOPANC, the aforementioned studies almost exclusively comprised patients in whom the liver was the site of the oligometastases, and who underwent hepatectomy accordingly. A small proportion of PDAC patients, however, have exclusive pulmonary metastases. There is some evidence regarding the outcome of lung resections in such a situation (Table 2).

Stuart et al. reported in a retrospective single-institution study on 39 patients with isolated lung recurrence after resection of the primary tumor [21]. A total of 14 of these patients underwent pulmonary metastasectomy without postoperative mortality and had a significantly longer median overall survival after recurrence than those who did not (30.8 vs. 18.6 months).

Ilmer et al. published a retrospective analysis from two centers comprising patients with metachronous lung metastases after resection of the primary [22]. Out of 15 patients undergoing planned pulmonary metastasectomy, 11 had confirmed PDAC metastases. There was no postoperative mortality. Median overall survival after diagnosis of metastatic disease was 26 months, and disease-free survival was 18 months. Longer metastasis-free interval (>17 months) and favorable tumor grade were associated with better outcomes.

The aforementioned analysis by Liu et al. conducted in the SEER database for the years 2010–2014 also included a subgroup of 740 patients with synchronous lung metastasis from PDAC. Of these, 21 underwent resection. Survival from diagnosis was 14.0 months versus 6.0 months for resected versus not resected patients (*p* < 0.0001) [17].

The hitherto largest series on patients with isolated pulmonary oligometastases from PDAC was published by Groot et al. [23]. It included 96 patients, out of whom 19 underwent metastasectomy. Median overall survival from initial resection was 39.6 months in the overall study population and 68.9 months in the metastasectomy subgroup. A recurrence-free interval >16 months prior to metastasectomy and active treatment (systemic therapy and/or metastasectomy) were associated with longer and >5 pulmonary lesions, symptoms at recurrence and a CA19-9 ≥ 100 U/mL with shorter survival.

The conversion rate, i.e., the proportion of oligometastatic patients who proceed to metastasectomy, is not consistently reported. Moreover, the potential denominator populations vary widely across studies. Where available, the conversion rate ranges from 2 to 44.5%. Postoperative morbidity is reported in almost all studies, but the definitions vary and not all studies distinguish between major and minor complications. The reported rates vary but generally are in the range of what is reported for hepatic and pulmonary resections in general.

The available evidence can be summarized as pointing towards a survival advantage for selected patients with PDAC and oligometastases who undergo staged or simultaneous resection of the primary tumor and the metastases. A longer time interval between resection of the primary and occurrence of metastases in metachronous cases, fewer metastases, lower levels of tumor markers, and the application and amount of systemic therapy were common factors associated with longer overall survival. Patients with isolated pulmonary oligometastases appear to form a distinct subgroup with a more favorable prognosis and, for PDAC, extraordinarily long overall survival after metastasectomy.

However, this presented evidence is exclusively retrospective, and selection of patients for the analyses in general and metastasectomy in particular, as well as the definition of oligometastatic disease, the used chemotherapy regimens, and the follow-up of patients are inevitably heterogenous across studies, making results highly prone to bias. Moreover, no study has reported quality-of-life results from validated tools (e.g., EORTC QLQ-C30, PAN26) which is a relevant gap in evidence. Consequently, there is a need for more unbiased data from prospective controlled trials, ideally randomized, assessing both survival and quality of life outcomes, and comparing different chemotherapy regimens, to answer the pertinent questions on the outcomes of metastasectomy in PDAC patients with oligometastases.

## 4. Ongoing Prospective Trials

There are five ongoing prospective trials for patients with oligometastatic PDAC (Table 3). The multicenter, prospective, randomized-controlled CSPAC-1 trial was initiated by the Chinese Study Group for Pancreatic Cancer to determine whether synchronous resection of the primary tumor and liver metastases following conversion chemotherapy can prolong survival compared with standard non-surgical treatment [24]. Patients eligible for the trial have no more than three hepatic metastases, without evidence of extrahepatic spread. All patients undergo first-line systemic chemotherapy as an initial selection phase. Only patients demonstrating stable disease or tumor regression and with favorable tumor marker dynamics proceed to the randomization phase. This enriches the study population for biologically favorable disease and mirrors real-world conversion strategies. Randomization is between simultaneous resection of the primary tumor and liver metastases or continuation of systemic therapy. Postoperative chemotherapy is recommended in the surgical group, while patients in the control group continue systemic treatment according to contemporary standards. The primary endpoint is overall survival from diagnosis, with secondary endpoints including overall survival from randomization, quality of life, and procedure-related morbidity and mortality. CSPAC-1 is powered to detect a clinically meaningful survival difference between the two treatment strategies and plans to randomize approximately three hundred patients from a much larger initial screening population undergoing induction chemotherapy.

Two prospective trials are currently conducted in or led from Germany. HOLIPANC is a multicenter, open-label trial designed to evaluate the feasibility, safety, and oncological potential of a neoadjuvant chemotherapy–surgery approach in patients with PDAC and no more than five synchronous liver metastases [25]. All patients receive standardized neoadjuvant combination chemotherapy. Patients with disease progression are excluded from further protocol treatment while patients with stable disease or tumor response are considered for synchronous resection when complete macroscopic tumor clearance appears technically achievable. The primary outcome is overall survival following complete tumor resection. Secondary outcomes include resection rates, patterns of disease recurrence, postoperative complications, and patient-reported quality of life.

METAPANC is an international randomized trial under the auspices of the German Working Group Medical Oncology (AIO) for patients with PDAC and no more than three synchronous or metachronous liver metastases [26]. Participants receive standardized induction chemotherapy prior to randomization, thereby ensuring that only patients with disease control and acceptable treatment tolerance proceed to treatment allocation. Subsequently, they are randomly assigned to either resection of the primary tumor and resection or ablation of the metastases, followed by postoperative chemotherapy, or to continuation of systemic chemotherapy without surgical intervention. Overall survival is the primary endpoint. Secondary endpoints include progression-free survival, postoperative morbidity and mortality, and quality of life.

SONAR is an Italian multicenter, randomized trial including patients with up to three hepatic metastases with a resectable or borderline resectable primary tumor [27]. All patients enrolled in the study must have completed a prolonged course of first-line systemic chemotherapy prior to randomization. Only patients demonstrating stable disease or objective response after at least six months of systemic therapy are considered eligible. Patients are then randomized to either surgical resection or continued non-surgical management, consisting of observation or continuation of chemotherapy according to institutional standards. The primary endpoint of SONAR is overall survival. Secondary endpoints include progression-free survival, perioperative morbidity and mortality, treatment-related toxicity, nutritional status, and patient-reported quality of life.

The PHOLIPANC trial (NCT06122480) is a single-arm phase II study enrolling patients with “limited” liver or lung metastases who have responded to four cycles of FOLFIRINOX induction chemotherapy. The primary endpoint is overall survival at two years. Secondary endpoints include progression-free survival, quality of life, and postoperative complications. The trial has completed enrolment and follow-up is ongoing.

## 5. Conclusions and Future Directions

Surgery for oligometastatic PDAC is a concept born out of the need for therapeutic improvement for patients with a limited prognosis. Biologically, building on the assumption of a truly oligometastatic state with limited systemic progression of PDAC, resection of remaining disease manifestations could provide an outlook for achieving longer-term remission. A surgical approach can, however, only be justified if postoperative outcomes in terms of morbidity, mortality, quality of life, and, importantly, the ability to receive more systemic therapy if required, are favorable.

The available pertinent evidence is exclusively from retrospective series. The results of most of these series are favorable with many of them demonstrating a longer survival in patients who underwent surgery versus systemic treatment without resection. Common positive predictors of survival are good imaging and tumor marker response to systemic therapy. Moreover, safety is good with acceptable morbidity and mortality rates. Metastatic site may play a role, with patients with isolated pulmonary oligometastases showing a better prognosis than those with hepatic metastases. Doubtless, this retrospective evidence is amenable to selection and publication bias, and the interpretability and implications of results are limited by the absence of standardized criteria for oligometastatic disease and the heterogeneity in selection criteria based on which patients are offered resection or not, in the sequence and characteristics of systemic regimens, and the surgical approaches. The true proportion of oligometastatic patients who proceed to surgical exploration and ultimately resection is not consistently reported across studies and probably not easily assessable at the single-center level given that many metastatic patients might not even be referred for evaluating surgery. The best estimates are available from population-based studies such as the SEER analysis by Liu et al. [17], which suggests that only a very small proportion of all metastatic patients become candidates for surgery. Moreover, observed survival benefits after surgery may in part reflect exceptional chemosensitivity rather than a direct effect of surgical tumor removal, making it difficult to isolate the independent contribution of surgery. Lastly, the external validity of existing data can be limited by their origin from high-volume pancreatic cancer centers. The results of the ongoing prospective non-randomized and randomized controlled trials are therefore eagerly awaited and will provide less biased and more standardized evidence on the topic.

At present, a number of limitations and unresolved issues prevent the routine implementation of surgery for oligometastatic PDAC in clinical practice. The principal challenge remains appropriate patient selection, as radiological response or stable disease after systemic therapy is an imprecise surrogate for favorable tumor biology, and even a substantial proportion of carefully selected patients still experience early recurrence after resection. In parallel, the concept of oligometastatic disease lacks a uniform definition, with considerable variability across studies regarding the number, size, and distribution of metastatic lesions, and uncertainty as to whether simple lesion counts adequately reflect true tumor biology. The optimal timing and duration of preoperative systemic therapy also remain unclear, as prolonged treatment may improve biological selection but risks cumulative toxicity and loss of resectability in case of a waning therapeutic effect; whereas shorter treatment intervals may lead to premature surgery in biologically aggressive disease. Although biomarkers and advanced imaging hold promise for improving biological stratification, their role in guiding clinical decision-making has not been fully established.

From a surgical perspective, combined pancreatic and hepatic resections are associated with meaningful morbidity, and the impact of postoperative complications on the delivery of further systemic therapy and on long-term oncological outcomes has not been fully elucidated. In patients with synchronous metastases, a choice between simultaneous pancreatic resection and metastasectomy and a staged approach needs to be made. Simultaneous resections carry a higher morbidity than either procedure alone [28], and a staged approach allows for biological assessment of the growth dynamics of the disease, possibly selecting patients with a more favorable tumor biology. On the other hand, there is the risk of progression of metastases during the interval between the two stages of surgery, rendering a resectable situation an irresectable one. There are no direct prospective comparisons between a one- and two-stage approach. Based on the available indirect evidence, it seems reasonable to perform simultaneous resection only in patients with appropriate performance status and smaller, favorably located hepatic metastases necessitating only minor hepatectomy. Moreover, a simultaneous resection is more justifiable in distal pancreatectomy compared to pancreatoduodenectomy, given the former has a lower morbidity risk. In this context, surgical access and technique may play an important role. Minimally invasive pancreatectomy and hepatectomy are associated with reduced morbidity and may thus lead to enhanced recovery and earlier resumption of chemotherapy [29,30]. So far, however, there are no studies specifically assessing minimally invasive resection for oligometastatic PDAC.

With the advent of potent systemic therapies for PDAC, surgeons sometimes face a situation of “disappearing metastasis”, where a formerly present lesion can hardly or not at all be visualized on imaging or intraoperatively. In the study by Frigerio et al. [13], complete disappearance of liver metastases was found in 4.5% of patients. The decision whether to resect the original metastatic bed, which may contain viable tumor cells, needs to be made. Intraoperative ultrasound and palpation can be valuable aids in identifying residual disease [31].

Recently, an important step towards standardization and consensus in the definition and management of patients with oligometastatic PDAC has been made. The OligoPanc project, an international and interdisciplinary expert panel, has issued a consensus statement regarding the most important aspects [32]. There, oligometastatic disease is defined as no more than three metastatic lesions in one organ, either the liver or the lung. It is concluded that patient-intrinsic factors are important for decision-making, which should take place in multidisciplinary tumor boards. Systemic treatment is declared mandatory before local treatment of the primary and the metastases. Metastasis-directed treatment should be considered, but there is no clear recommendation for such treatment in all oligometastatic patients, and no specific modality of treatment is recommended.

Surgery for oligometastatic PDAC competes with other metastasis-directed treatments such as stereotactic radiation or ablation. In the multicenter randomized phase II XTEND trial, 41 patients with PDAC and ≤5 metastatic lesions were randomized to receive chemotherapy alone or chemotherapy plus metastasis-directed treatment which almost exclusively consisted of stereotactic body radiotherapy [33]. The primary endpoint progression-free survival was 10.3 months with chemotherapy plus metastasis-directed treatment compared to 2.5 months with chemotherapy alone. Importantly, no grade ≥3 toxicities attributable to metastasis-directed treatment were observed. The ongoing phase III EXPAND trial is designed as a confirmatory study for the concept (NCT06593431). There are no completed or ongoing trials directly comparing surgery with radiotherapy for oligometastatic PDAC, so a conclusive statement which of the two modalities is more favorable is not possible.

**Figure 1:**
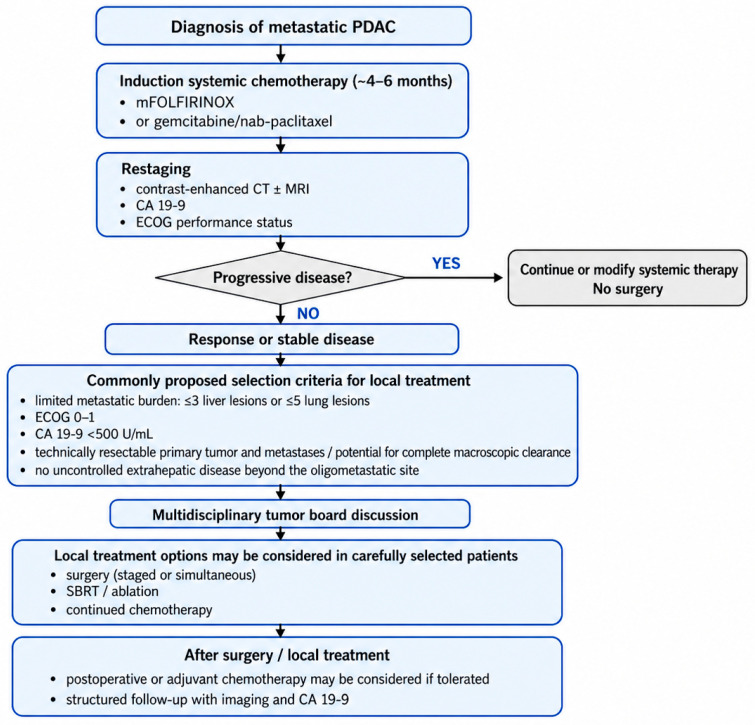
Clinical algorithm for the management of patients with oligometastatic PDAC. ECOG: Eastern Cooperative Oncology Group. SBRT: Stereotactic Body Radiation Therapy.

Individual and shared decision-making is crucial in patients with oligometastatic PDAC. Nonetheless, taking into account the presented evidence, a clinical algorithm for their management can be proposed. It incorporates longer-term chemotherapy prior to deciding about resection based on the results of imaging and serological and clinical re-staging and gives the choice between surgery and ablation as local therapy (Figure 1).

Future progress in the management of oligometastatic pancreatic cancer will depend on a more refined integration of surgery, local ablative therapies, and biology-driven patient selection. Surgical resection is likely to evolve from an all-or-nothing strategy toward a more individualized component of multimodal treatment, reserved for patients in whom durable disease control with systemic therapy and favorable biological behavior can be demonstrated. In this context, local therapies such as thermal ablation or stereotactic body radiotherapy (SBRT) may complement or, in selected cases, substitute surgical resection to achieve local disease control while minimizing procedural morbidity, particularly in patients with limited hepatic involvement or marginal surgical fitness. An important future direction lies in the incorporation of liquid biopsy-based approaches, including circulating tumor DNA (ctDNA) and other blood-based biomarkers, to improve patient selection beyond conventional imaging and clinical criteria. ctDNA has shown good prognostic properties in the neoadjuvant setting for localized PDAC and in the metastatic setting but remains to be evaluated in oligometastatic stages [34,35]. Dynamic assessment of circulating tumor burden during systemic therapy may allow earlier identification of patients with indolent disease biology and sustained molecular response, as well as of those with occult systemic progression. Combined with advanced imaging and multidisciplinary evaluation, such biologically informed strategies may enable a more precise allocation of surgical and local treatments, ultimately shifting surgery toward a personalized consolidative approach within a broader, biology-driven treatment algorithm.

## Figures and Tables

**Table 1:** Summary of clinical studies evaluating surgical approaches for oligometastatic PDAC. Major morbidity: Clavien–Dindo III-IV complications and/or pancreatic fistula grade B/C, post-hepatectomy liver failure, reoperation.

First Author (Year)	Patients Resected/Total (*n*), Conversion Rate (%) ^1^	Metastases	Preoperative Systemic Therapy	OS (Definition)	PFS (Definition)	Postop. Overall/Major Morbidity	Postop. Mortality	Predictors of Longer Survival
Shrikhande (2007) [11]	29/193, 15.0%	Synchronous liver, interaortocaval or peritoneal metastases (incidental intraoperative finding)	3% of patients (chemoradio-therapy)	Median OS 13.8 mo from surgery	NR	24.1%/NR	0%	Interaortocaval metastatic site, adjuvant chemotherapy
Tachezy (2016) [12]	NR/69	Synchronous liver metastases	14% of patients	Median OS 14.5 mo from surgery	NR	68%/8%	1.4%	Resection status
Frigerio (2017) [13]	24/535, 4.5%	Initially synchronous liver metastases; disappeared after chemotherapy	100% of patients	Median OS 56 mo from diagnosis (28 mo from surgery)	Median DFS 27 mo from surgery (13 mo from surgery)	66%/NR	0%	None
Hackert (2017) [14]	128/NR	Liver or distant interaortocaval lymph nodes	NR	Median OS 12.3 mo from surgery	NR	45%/NR	2.9%	Limited metastases; R0 resection
Hank (2023) [15]	93/173, 53.8% (denominator: patients who underwent exploration, total number of metastatic patients NR)	Liver, peritoneum, distant lymph nodes	100% of patients	Median OS 25.5 mo (ypM0), 10.7 mo (ypM1), 8.1 mo without resection	NR	NR/19.4%	3.2%	ypM0, timing of resection, CA 19-9
Kandel (2018) [16]	6/NR	Liver, lung	100% of patients	Median OS 32.4 mo (resected patients)	NR	NR	NR	Resection
Liu (2020) [17]	128/6252, 2.0%	Liver	NR	10.0 mo (from surgery), 4.0 mo (without resection)	NR	NR	NR	Resection
Bachellier (2022) [18]	92/NR	Synchronous liver metastases	100% of patients	18.3 mo (from diagnosis), 12.7 mo (from surgery)	NR	40.2%/37%	5.4%	CA 19-9, R0 resection, adjuvant chemotherapy
Nagai (2023) [19]	47/NR	Liver metastases	68% of patients	21.9 mo (from diagnosis)	6.1 mo (from surgery)	45%/17%	0%	Preoperative chemotherapy, differentiation of the primary tumor
Parent (2026) [20]	69/155, 44.5%	Liver, lung, other	NR	3.4 mo	0.8 mo	NR	NR	CA 19-9, number and location of metastasis

^1^ Conversion rate: Proportion of all oligometastatic patients in the respective study who undergo metastasectomy. Abbreviations: OS = overall survival; PFS = progression-free survival (as defined in the single study); DFS = disease-free survival (as defined in the single study); mo = months; NR = not reported; PDAC = pancreatic ductal adenocarcinoma; R0 = microscopically margin-negative resection.

**Table 2:** Summary of clinical studies evaluating surgical approaches for isolated pulmonary oligometastatic PDAC. Major morbidity: Clavien–Dindo III-IV complications and/or pancreatic fistula grade B/C, post-hepatectomy liver failure, reoperation.

First Author (Year)	Patients Total/Resected (*n*), Conversion Rate (%) ^1^	Metastases	Preoperative Systemic Therapy	Median OS (Definition)	Median PFS (Definition)	Postop. Overall/Major Morbidity	Postop. Mortality	Predictors of Longer Survival
Stuart (2023) [21]	14/39, 35.8%	Metachronous isolated pulmonary metastases	43%	Mean OS: 52.7 mo (from initial pancreatectomy), 30.8 mo (from pulmonary metastasectomy)	NR	14%/7%	0%	Metastasectomy
Ilmer (2019) [22]	11/13, 84.6% (denominator: patients who underwent exploration, total number of metastatic patients NR)	Metachronous isolated pulmonary metastases	None after adjuvant therapy after primary pancreatectomy	43 mo (from initial pancreatectomy), 26 mo (from pulmonary metastasectomy)	NR	9%/0%	0%	Disease-free interval after primary pancreatectomy, tumor grading
Liu (2020) [17]	21/740, 2.8%	Synchronous isolated pulmonary metastases	NR	14.0 mo (from surgery), 6.0 mo (without resection)	NR	NR	NR	Resection
Groot (2019) [23]	19/96, 19.8%	Metachronous isolated pulmonary metastases	None after adjuvant therapy after primary pancreatectomy	68.9 mo (from initial pancreatectomy), 27.1 mo (from lung metastasectomy)	35.0 mo (from lung metastasectomy)	15.8%/0%	0%	Recurrence-free interval, metastasectomy, number of lesions, CA 19-9

^1^ Conversion rate: Proportion of all oligometastatic patients in the respective study who undergo metastasectomy. Abbreviations: OS = overall survival; PFS = progression-free survival (as defined in the single study); PDAC = pancreatic ductal adenocarcinoma.

**Table 3:** Summary of ongoing controlled trials for oligometastatic PDAC.

Study Identifier	Main Inclusion Criteria	Sample Size	Study Treatments	Primary Endpoint	Secondary Endpoints
CSPAC-1—NCT03398291 [23]	PDAC with ≤3 synchronous liver metastases after response to induction chemotherapy	300	Induction chemotherapy (FOLFIRINOX or gemcitabine-based) → simultaneous resection + adjuvant therapy vs. chemotherapy alone	OS	PFS; postoperative morbidity/mortality; QoL; R0 resection
METAPANC—EuCT Nr 2023-503558-10-00 [24]	Resectable PDAC with ≤3 synchronous or metachronous hepatic metastases, randomization after response to induction chemotherapy	272	Induction mFOLFIRINOX → resection of primary tumor and liver metastases vs. chemotherapy continuation	OS	PFS; safety; QoL; R0 resection
HOLIPANC—NCT04617457 [25]	PDAC with ≤5 synchronous liver metastases	150	Neoadjuvant NALIRIFOX (liposomal irinotecan + oxaliplatin + 5-FU/leucovorin) → simultaneous resection (ablation of liver metastases possible)	OS after R0/R1 resection	OS of all patients; PFS; R0/R1 resection; toxicity; postoperative morbidity
SONAR NCT06690528 [26]	PDAC with ≤3 synchronous liver metastases after response to induction chemotherapy	56	Induction chemotherapy (FOLFIRINOX or gemcitabine/nab-paclitaxel) → surgery vs. systemic therapy	OS at 2 years	PFS; QoL; postoperative morbidity
PHOLIPANC—NCT06122480	PDAC with “limited” synchronous pulmonary or hepatic metastases after response to induction chemotherapy	40 (completed)	Resection of primary tumor and metastases	OS at 2 years	PFS; QoL; postoperative morbidity

Abbreviations: OS = overall survival; PFS = progression-free survival; PDAC = pancreatic ductal adenocarcinoma; R0 = microscopically margin-negative resection.

## Data Availability

The data presented in this study are available in and were extracted from the respective original publications. No individual patient data were used.

## Supplementary Materials

The following supporting information can be downloaded at: https://www.mdpi.com/article/10.3390/cancers18111699/s1, Table S1: Quality and risk-of-bias assessment of the single included studies using the Newcastle-Ottawa Scale (NOS).
