# Supplementary material for: Surgery in Oligometastatic Pancreatic Cancer: Narrative Systematic Review
Source: Cancers (Basel). 2026 May 23;18(11):1699. doi: 10.3390/cancers18111699 (PMC13256052; doi:10.3390/cancers18111699)
Supplement: Supplementary file 1 [file cancers-18-01699-s001.zip › cancers-4253728-supplementary.pdf]

Table S1: Quality and risk-of-bias assessment of the single included studies using the Newcastle-Ottawa Scale (NOS).

Study	Representativeness of exposed cohort	Selection of non-exposed cohort	Ascertainment of exposure	Outcome not present at start	Comparability of cohorts	Assessment of outcome	Follow-up long enough	Adequacy of follow-up	Total NOS score
Shrikhande (2007) [11]	–	★	★	★	–	★	–	★	5
Tachezy (2016) [12]	–	–	★	★	–	★	★	★	5
Frigerio (2017) [13]	★	★	★	★	–	★	★	★	7
Hackert (2017) [14]	–	–	★	★	–	★	–	–	3
Hank (2023) [15]	★	★	★	★	–	★	★	★	7
Kandel (2018) [16]	–	–	★	★	–	–	–	–	2
Liu (2020) [17]	★	★	★	★	–	★	★	★	7
Bachellier (2022) [18]	–	–	★	★	–	★	–	★	4
Nagai (2023) [19]	–	–	★	★	–	★	★	★	5

Study	Representativeness of exposed cohort	Selection of non-exposed cohort	Ascertainment of exposure	Outcome not present at start	Comparability of cohorts	Assessment of outcome	Follow-up long enough	Adequacy of follow-up	Total NOS score
Parent (2026) [20]	★	★	★	★	★	★	–	–	6
Stuart (2023) [21]	★	★	★	★	★	★	★	–	7
Ilmer (2019) [22]	–	–	★	★	–	★	★	–	4
Groot (2019) [23]	★	★	★	★	★	★	★	–	7
